# Myelination of Neuronal Cell Bodies when Myelin Supply Exceeds Axonal Demand

**DOI:** 10.1016/j.cub.2018.02.068

**Published:** 2018-04-23

**Authors:** Rafael G. Almeida, Simon Pan, Katy L.H. Cole, Jill M. Williamson, Jason J. Early, Tim Czopka, Anna Klingseisen, Jonah R. Chan, David A. Lyons

**Affiliations:** 1 Centre for Discovery Brain Sciences, University of Edinburgh, 49 Little France Crescent, Edinburgh EH16 4SB, UK; 2 Department of Neurology and Program in Neuroscience, University of California, San Francisco, 675 Nelson Rising Lane, San Francisco, CA 94143, USA; 3 Institute of Neuronal Cell Biology, Technical University of Munich, Biedersteiner Strasse 29, 80802 Munich, Germany; 4 Munich Cluster of Systems Neurology (SyNergy), Feodor-Lynen Strasse 17, 81377 Munich, Germany

**Keywords:** myelin, oligodendrocyte, neuron, axon, myelination, CNS, myelin mistargeting, zebrafish

## Abstract

The correct targeting of myelin is essential for nervous system formation and function. Oligodendrocytes in the CNS myelinate some axons, but not others, and do not myelinate structures including cell bodies and dendrites [1]. Recent studies indicate that extrinsic signals, such as neuronal activity [2, 3] and cell adhesion molecules [4], can bias myelination toward some axons and away from cell bodies and dendrites, indicating that, *in vivo*, neuronal and axonal cues regulate myelin targeting. *In vitro*, however, oligodendrocytes have an intrinsic propensity to myelinate [5, 6, 7] and can promiscuously wrap inert synthetic structures resembling neuronal processes [8, 9] or cell bodies [4]. A current therapeutic goal for the treatment of demyelinating diseases is to greatly promote oligodendrogenesis [10, 11, 12, 13]; thus, it is important to test how accurately extrinsic signals regulate the oligodendrocyte’s intrinsic program of myelination *in vivo*. Here, we test the hypothesis that neurons regulate myelination with sufficient stringency to always ensure correct targeting. Surprisingly, however, we find that myelin targeting *in vivo* is not very stringent and that mistargeting occurs readily when oligodendrocyte and myelin supply exceed axonal demand. We find that myelin is mistargeted to neuronal cell bodies in zebrafish mutants with fewer axons and independently in drug-treated zebrafish with increased oligodendrogenesis. Additionally, by increasing myelin production of oligodendrocytes in zebrafish and mice, we find that excess myelin is also inappropriately targeted to cell bodies. Our results suggest that balancing oligodendrocyte-intrinsic programs of myelin supply with axonal demand is essential for correct myelin targeting *in vivo* and highlight potential liabilities of strongly promoting oligodendrogenesis.

## Highlights

• Balance between axons and myelin production regulates its targeting *in vivo*
• Excess myelin is mistargeted to cell bodies
• Low, but not zero, level of mistargeting during normal development
• Potential implications for myelin-promoting therapies

## Graphical Abstract

**Figure:**
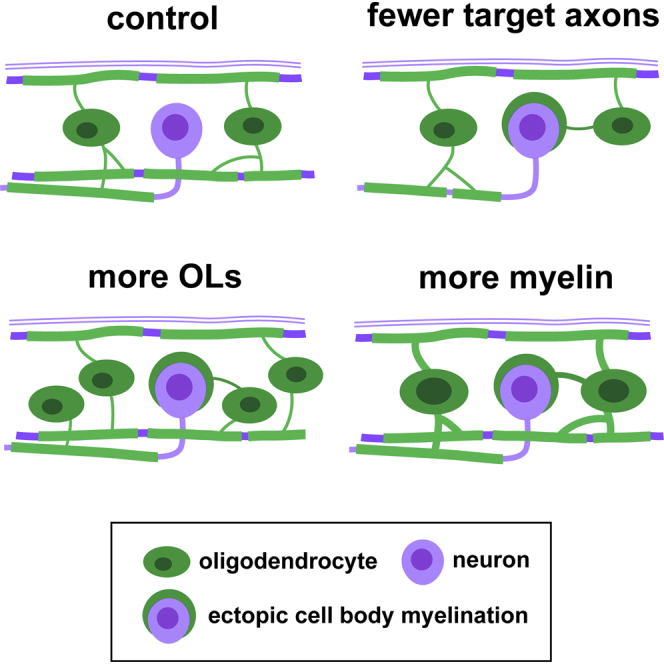

## Teaser

Almeida et al. find that the balance between axon demand and myelin production in the CNS regulates myelin targeting. In the developing CNS, when normal target axons are reduced, oligodendrocyte number increased, or myelin production per oligodendrocyte increased, myelin is mistargeted to cell bodies, including those of neurons.

## Results and Discussion

### Oligodendrocytes Mistarget Myelin to Neuron Cell Bodies when Target Axon Number Is Reduced

**Figure 1:**
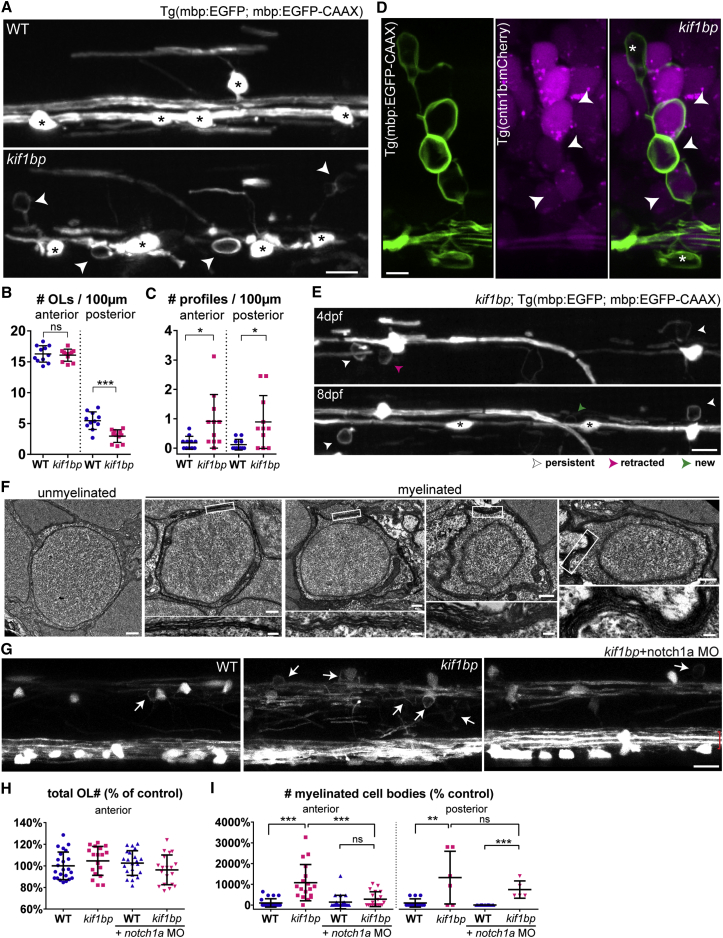
Oligodendrocytes in Excess of Target Axons Ectopically Myelinate Neuronal Cell Bodies in *kif1bp* Mutants (A) Double myelin and oligodendrocyte reporter showing oligodendrocytes (asterisks) and ectopic profiles (arrowheads) in the spinal cord (4 dpf). (B) Oligodendrocyte number (per 100 μm length of spinal cord) is normal in the anterior but reduced in the posterior spinal cord of *kif1bp* mutants (p = 0.688 anterior; p = 0.0001 posterior; n = 11 wild-type [WT] and n = 11 mutants; t test). (C) Profile number is increased in mutants (p = 0.018 anterior; p = 0.011 posterior; n = 11 WT and n = 11 mutants; t test). (D) Double myelin and neuronal reporter showing mCherry^+^ neurons enwrapped by EGFP+ myelin (arrowheads) in 4 dpf mutants. Asterisk, oligodendrocyte cell body. (E) Time course of double oligodendrocyte and myelin reporter in *kif1bp* mutant. Asterisks denote new oligodendrocytes. (F) Transmission electron microscopy (TEM) of a typical unmyelinated cell body and four myelinated cell bodies in 6 dpf mutants. (G) 4 dpf anterior spinal cord of oligodendrocyte and myelin reporter showing that increased ectopic profiles (arrows) in mutants are rescued by presence of additional Mauthner axons (red brackets) when injected with *notch1a* morpholino (MO). (H) Oligodendrocyte number (normalized to control average) is normal in all conditions (p_[kif1bp-WT]_ = 0.267; p_[WT+notch1a MO-WT]_ = 0.483; p_[kif1bp+notch1aMO-WT]_ = 0.374; p_[kif1bp+notch1aMO-kif1bp]_ = 0.071; n = 23 WT; n = 18 *kif1bp*; n = 22 WT+notch1a MO; n = 19 *kif1bp*+notch1a MO; t tests). (I) Myelinated cell body number (normalized to control average) is increased in mutants (p_[WT-kif1bp]_ < 0.0001, n = 23 WT and n = 18 *kif1bp* anterior; p_[WT-kif1bp]_ = 0.0019, n = 14 WT and n = 6 *kif1bp* posterior; t tests) and is rescued by additional Mauthner axons in anterior (p_[kif1bp+notch1a MO-kif1bp]_ = 0.0008, n = 18 *kif1bp* and n = 19 *kif1bp*+notch1a MO; t test), but not posterior, spinal cord of mutants (p_[kif1bp+notch1a MO-kif1bp]_ = 0.359, n = 6 *kif1bp* and n = 5 *kif1bp*+notch1a MO; t test). All graphs display mean and SD. OL, oligodendrocyte. The scale bars represent 10 μm (A, E, and G), 5 μm (D), 0.5 μm (F, whole cell), and 0.1 μm (F, myelin detail). See also Movies S1 and S2.

Oligodendrocytes target myelin promiscuously *in vitro* [4, 8, 14], but *in vivo*, CNS myelin is selectively targeted to specific axons and not to dendrites or cell bodies [1]. Our previous live-imaging studies indicate that oligodendrocytes can retract newly formed myelin sheaths [15] and that axons can regulate the myelinating capacity of oligodendrocytes [16]. These observations led us to hypothesize that axons would always regulate myelination with sufficient stringency to ensure correct targeting. To test this prediction, we first analyzed myelination in an environment with a reduced number of target axons. Reticulospinal axons, the first myelinated in the zebrafish CNS, do not elongate completely along the ventral spinal cord of *kif1-binding protein* (*kif1bp*) mutants [17], leading to a 50%–80% reduction of reticulospinal axonal surface in the anterior and posterior spinal cord, respectively [18]. However, *kif1bp* mutants have a normal number of oligodendrocytes in the anterior spinal cord and about 54% of oligodendrocytes in the posterior (Figures 1A and 1B), creating a surplus of oligodendrocytes relative to target axons.

To analyze myelin targeting in *kif1bp* mutants, we crossed the myelin reporter line Tg(mbp:EGFP-CAAX) with the Tg(mbp:EGFP) line, in which all myelinating glia express cytoplasmic EGFP [16]. Strikingly, we observed unusual circular profiles in addition to normal-appearing myelin sheaths throughout the mutant spinal cord at 4 days post-fertilization (dpf) (Figures 1A and 1C), To test the possibility that these structures represented ectopically myelinated cell bodies, we crossed the Tg(mbp:EGFP-CAAX) line with the Tg(cntn1b:mCherry) line, in which many spinal neurons express cytoplasmic mCherry [15]. In *kif1bp* mutants, we readily identified mCherry-positive neuron cell bodies that were ectopically wrapped by EGFP-CAAX-positive oligodendrocyte membrane (Figure 1D; Movie S1). To further characterize these ensheathing profiles, we carried out electron microscopy of *kif1bp* mutants and readily identified cell bodies enwrapped with myelin-like membrane (Figure 1F). Thus, contrary to our prediction, when oligodendrocytes are present in excess of axons, they mistarget myelin to neuronal cell bodies, suggesting that myelin targeting is not always stringently regulated.

### Incomplete Correction of Myelin Mistargeting in *kif1bp* Mutants

We previously showed that individual oligodendrocytes initiate formation of all their myelin sheaths within ∼5 hr [15]. During this period, myelinating processes survey the local environment and decide which axons to myelinate and which structures to avoid. We performed time-lapse imaging of *kif1bp* mutants during myelin sheath formation, which revealed that the wrapping of cell bodies occurs concomitantly with the formation of normal sheaths by oligodendrocytes (Movie S2). After this dynamic period, individual oligodendrocytes can retract sheaths over the following days, allowing for refinement and correction of targeting errors that may have occurred [15]. Therefore, we followed the fate of wrapped cell bodies from 4 to 8 dpf (Figure 1E). In wild-types, we found that 11 out of the 13 profiles observed in 10 animals (sampled from a 900-μm-long stretch of each spinal cord) at 4 dpf were retracted by 8 dpf (Figure 1E). Interestingly, in *kif1bp* mutants, where we observed 78 profiles in 11 animals, 21 were retracted by 8 dpf, but this slightly higher rate of retraction was only able to correct 27% (21/78) of mistargeted profiles (Figure 1E). Thus, despite some correction of mistargeting, this is insufficient to correct abundant errors in *kif1bp* mutants. As more oligodendrocytes differentiate, more myelinated cell bodies emerge in mutants (49 profiles added between 4 and 8 dpf in 11 mutants; Figure 1E), whereas this remains rare in control (4 added between 4 and 8 dpf), highlighting how tightly regulated myelin targeting is during normal myelination.

Together, these data suggest that oligodendrocytes have a drive to produce myelin *in vivo* as *in vitro*, even with fewer target axons, which can result in myelin mistargeting to neuronal cell bodies.

### Increasing Target Axon Number Rescues the Wrapped Cell Body Phenotype of *kif1bp* Mutants

We reasoned that, if cell body wrapping is due to an excess of oligodendrocytes relative to target axons, it should be rescued by increasing target axon number in *kif1bp* mutants. To test this, we injected a morpholino to transiently downregulate *notch1a* expression, which generates supernumerary Mauthner neurons possessing very large caliber target axons in the spinal cord [16] (Figure 1G). This manipulation did not change oligodendrocyte number (Figure 1H), effectively reducing the relative discrepancy between oligodendrocyte number and axonal surface in *notch1a* morpholino-injected *kif1bp* mutants compared to uninjected mutants with just two Mauthner axons. The number of wrapped cell bodies was restored to wild-type levels in the anterior spinal cord of mutants with additional Mauthner axons (Figures 1G and 1I). Importantly, this rescue did not occur in the posterior spinal cord of *kif1bp* mutants, into which supernumerary Mauthner axons fail to grow, ruling out the possibility of a direct role of Notch1a signaling in rescuing this phenotype (Figure 1I). These results confirm that it is the mismatch between target axons and oligodendrocyte number—and not disruption of *kif1bp*—that causes the ectopic wrapping of cell bodies.

### Myelin Is Not Mistargeted to Small-Caliber Unmyelinated Axons in *kif1bp* Mutants

**Figure 2:**
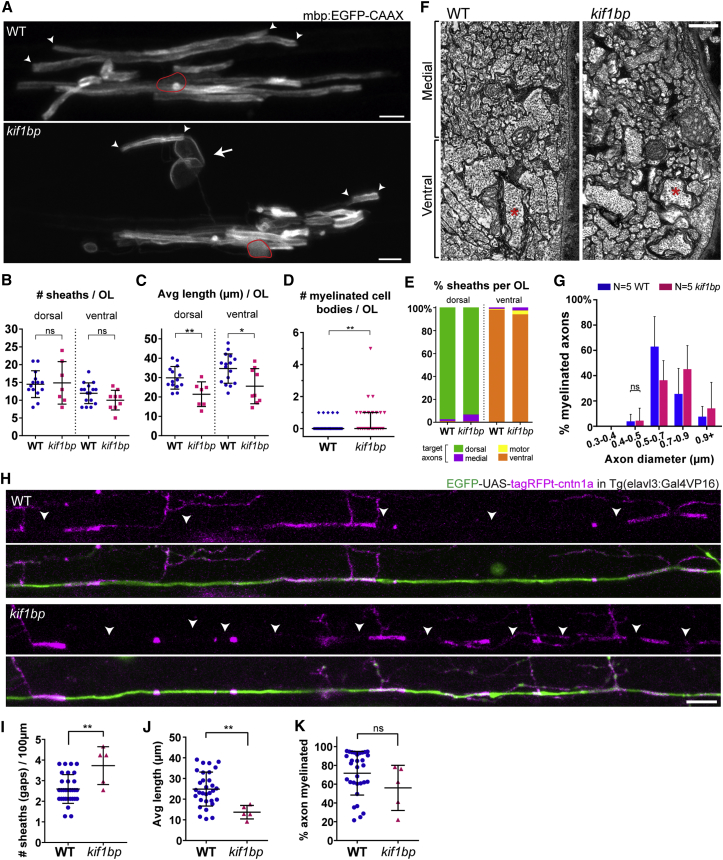
Individual Oligodendrocytes in Excess of Target Axons Wrap Cell Bodies and Normal Axon Targets, but Not Incorrect Axons (A) Individual oligodendrocytes (mbp:EGFP-CAAX^+^) in the 4 dpf anterior spinal cord. Arrow, myelinated cell body; arrowheads, normal myelin sheaths; red outline, oligodendrocyte cell body. (B) Sheath number per oligodendrocyte is normal (p = 0.868 dorsal, n = 14 WT and n = 7 mutants; p = 0.118 ventral, n = 16 WT and n = 9 mutants; t tests). (C) Average sheath length per oligodendrocyte is reduced in mutants (p = 0.007 dorsal, n = 14 WT and n = 7 mutants; p = 0.013 ventral, n = 16 WT and n = 9 mutants; t tests). (D) Number of myelinated cell bodies per oligodendrocyte is increased in mutants (p = 0.009, n = 35 WT and n = 25 mutants; Mann-Whitney test). (E) Proportion of myelin sheaths target axonal tracts in mutants is comparable to wild-type. (F) TEM at the onset of myelination (3.5 dpf) focusing on the ventral region with reticulospinal axons (e.g., asterisks) and adjacent medial region with small-diameter (<0.3 μm) unmyelinated axons. (G) The smallest myelinated axons remain above 0.4 μm diameter (p_[0.4–0.5μm]_ = 0.908; p_[0.5–0.7]_ = 0.251; p_[0.7–0.9]_ = 0.386; p_[0.9+]_ = 0.782; n = 5 WT and n = 5 mutants; corrected t tests). (H–J) Single EGFP+ reticulospinal axons (H) co-expressing the myelination reporter tagRFPt-cntn1a showing more and shorter tagRFPt-cntn1a gaps (i.e., myelin sheaths, arrowheads) in the anterior spinal cord of mutants at 4 dpf, quantified in (I) (p = 0.003; n = 31 WT and n = 5 mutants; t test), and (J) (p = 0.005; n = 31 WT and n = 5 mutants; t test). (K) % axon myelinated (tagRFPt-cntn1a negative) is comparable to wild-types (p = 0.174; n = 31 WT and n = 5 mutants; t test). All graphs display mean and SD, except (D) (median and interquartile range). The scale bars represent 5 μm (A), 0.5 μm (F), and 10 μm (H).

To further assess myelin targeting in *kif1bp* mutants, we analyzed individual mbp:EGFP-CAAX-expressing oligodendrocytes in the anterior spinal cord (Figure 2A). As expected, individual oligodendrocytes in mutants myelinated cell bodies more frequently than wild-type (11/29 cells in mutant animals versus 5/43 in wild-type; Figure 2D; Fisher exact test; p = 0.0187). Interestingly, individual oligodendrocytes made a normal number of sheaths in *kif1bp* mutants (Figure 2B), further suggesting that oligodendrocytes can make myelin sheaths independently of precise axonal demand. We found that the sheaths made by oligodendrocytes in *kif1bp* mutants were targeted to the axonal tracts that they typically myelinate, i.e., the dorsal tract for dorsal oligodendrocytes and the ventral tract for ventral oligodendrocytes (Figure 2E). Electron microscopy analyses of myelination in *kif1bp* mutants indicated that there was no observable increase in the myelination of inappropriate small-caliber axons (Figures 2F and 2G) and that myelin was restricted to the large-caliber (>0.4 μm diameter) axons that remained (Figures 2F and 2G). If most myelin is targeted to the remaining large-caliber axons in *kif1bp* mutants, we would expect to see more sheaths along individual axons and that such sheaths would be shorter in length. To test this prediction, we assessed myelination along individual reticulospinal axons using the tagRFPt-cntn1a reporter, which is initially localized along the entire axolemma but becomes excluded from regions with myelin sheaths [3]. We co-expressed cytoplasmic EGFP and tagRFPt-cntn1a in individual reticulospinal axons (see STAR Methods) and found that reticulospinal axons in *kif1bp* mutants had significantly more tagRFPt-cntn1a gaps (sheaths), which were shorter in length (Figures 2I–2K). In addition, our analysis of single-oligodendrocyte morphology indicated that the average length of myelin sheaths made per cell was significantly shorter in *kif1bp* mutants (Figure 2C). Although we cannot rule out an oligodendrocyte-autonomous role for *kif1bp* in regulating myelin sheath length, our data indicate that the availability of axonal space regulates correct myelin targeting. Our data suggest that oligodendrocytes target myelin in a hierarchical manner, first to large-caliber axons, as one would predict, and then, surprisingly, to cell bodies, which are more readily myelinated than inappropriate small-diameter axons.

### Myelin Is Also Mistargeted to Neuronal Cell Bodies in Animals with Increased Oligodendrocyte Number

**Figure 3:**
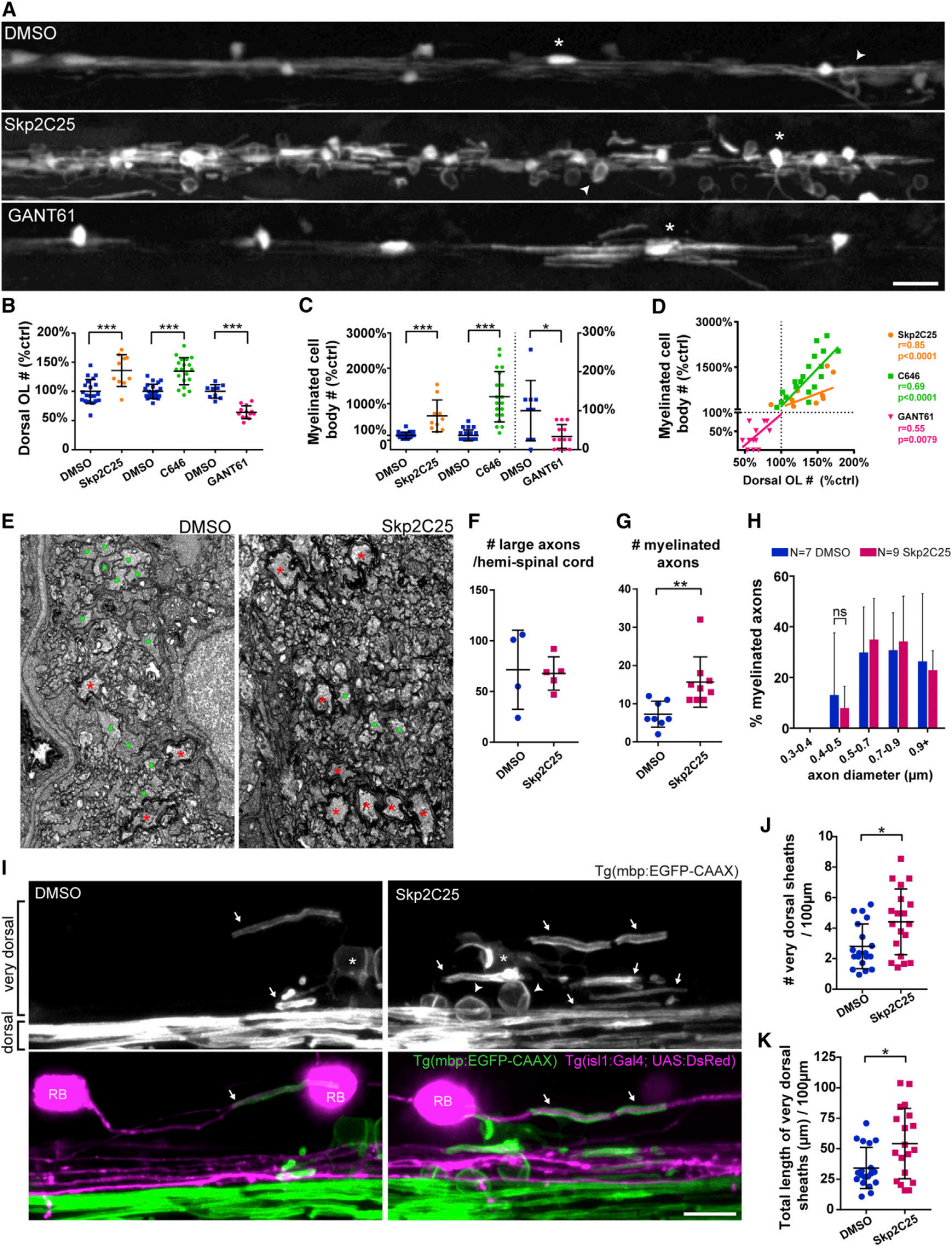
Increasing Oligodendrocyte Number Recapitulates Wrapping of Neuronal Cell Bodies (A) Double myelin and oligodendrocyte reporter (4 dpf), treated with vehicle DMSO, Skp2C25, or GANT61. (B and C) Oligodendrocyte (e.g., asterisk) number and myelinated cell bodies (e.g., arrowheads) number is increased in the dorsal spinal cord of Skp2C25-treated animals and reduced in GANT61-treated animals, quantified in (B) (p_[Skp2C25-DMSO]_ = 0.0004, n = 20 DMSO and n = 10 Skp2C25; p_[C646-DMSO]_ < 0.0001, n = 22 DMSO and n = 19 C646; p_[GANT61-DMSO]_ < 0.0001, n = 9 DMSO and n = 13 GANT61; t tests; data normalized to control average) and in (C) (p_[Skp2C25-DMSO]_ < 0.0001, n = 20 DMSO and n = 10 Skp2C25; p_[C646-DMSO]_ < 0.0001, n = 22 DMSO and n = 19 C646; p_[GANT61-DMSO]_ = 0.011, n = 9 DMSO and n = 13 GANT61; t tests; data normalized to control average; GANT61 data and its control plotted on right y axis), respectively. (D) Plotting all normalized data shows that myelinated cell body number positively correlates to oligodendrocyte number (Pearson’s *r* correlation and p value displayed for each treatment). (E) TEM of 4 dpf medial-dorsal spinal cord shows increased number of myelinated axons (red asterisks) in Skp2C25-treated animals whereas controls still have many unmyelinated large-caliber axons (green circles). (F) Total number of large-caliber (>0.5 μm diameter) axons in the medial-dorsal spinal cord is normal in Skp2C25-treated animals (p = 0.852; n = 4 DMSO and n = 5 Skp2C25; t test). (G) Myelinated axon number is increased with Skp2C25 (p = 0.006; n = 8 DMSO and n = 9 Skp2C25; t test). (H) Smallest myelinated axons in Skp2C25-treated animals remain above 0.4 μm diameter (p = 0.961 for all bin comparisons; n = 7 DMSO and n = 9 Skp2C25; corrected t tests). (I) 4 dpf double myelin and Rohon-Beard (RB) neuron reporter (Tg(isl1(ss):Gal4; UAS:DsRed)), focusing in the dorsal spinal cord. (J and K) Skp2C25-treated animals have more “very dorsal” myelin sheaths (arrows), quantified in (J) (p = 0.011; n = 19 DMSO and n = 19 Skp2C25; t test), which add up to a higher total myelin sheath length, quantified in (K) (p = 0.013; n = 19 DMSO and n = 19 Skp2C25; t test), as well as readily identifiable myelinated cell bodies (arrowheads). Examples of very rare myelin sheaths on dsRed+ large-caliber RB axons (arrows in merged panels), which are not increased with Skp2C25 (see main text for details), are shown. All graphs display mean and SD. The scale bars represent 20 μm (A) and 10 μm (I). See also Figure S1.

There is currently a major drive to identify compounds that promote oligodendrocyte differentiation *in vivo*, given that a bottleneck in efficient oligodendrocyte differentiation is thought to limit myelin regeneration in multiple sclerosis [10, 11, 12]. Chemical screens have recently identified several compounds that promote oligodendrocyte differentiation and remyelination in both toxin and inflammatory models of demyelination [19, 20, 21, 22, 23]. We recently completed an analogous chemical discovery screen in zebrafish that identified further compounds, including Skp2C25 and C646, inhibitors of Skp2 and p300/CBP, respectively, that promote oligodendrogenesis (J.J.E., K.L.H.C., J.M.W., H. Kamadurai, M. Muskavitch, D.A.L., unpublished data). Treatment with 2 μM Skp2C25 or 2 μM C646 between 2 and 4 dpf (i.e., during oligodendrocyte development and after most neurogenesis) leads to an increase in myelinating oligodendrocyte number (Figures 3A and 3B). Electron microscopy characterization of the effects of Skp2C25 treatment showed that the increase in oligodendrocytes results in an increase in myelinated axons, without affecting axonal number or size (Figures 3E–3H). These compounds allowed us to independently test whether increasing oligodendrocyte number relative to target axons leads to myelin mistargeting. We quantified myelinated cell bodies as before and found more wrapped cells in both Skp2C25- and C646-treated animals (Figures 3A and 3C), many of which are neuronal, as seen in *kif1bp* mutants (see Figure S1). Interestingly, the number of wrapped cell bodies correlated positively with the number of oligodendrocytes in individual animals, whereby individual animals with a larger increase in oligodendrocytes had a much greater increase in myelinated cell bodies (Figure 3D). Potentially, a slight increase in oligodendrocyte number may lead to the production of sufficient myelin to saturate all available myelination-competent axonal space, a consequence of which would be that a much larger increase in oligodendrocyte number would lead to a proportionally greater mistargeting of myelin to cell bodies.

We next asked whether excess myelin in Skp2C25-treated animals, observed by both electron (Figures 3E and 3G) and light (Figures 3I–3K) microscopy, was solely targeted to the correct axons (precociously) and inappropriately to cell bodies. Similar to *kif1bp* mutants, we found that axons smaller than 0.4 μm in caliber remained unmyelinated in Skp2C25-treated animals (Figures 3E and 3H). Indeed, biophysical constraints can regulate myelination: oligodendrocytes readily myelinate inert fibers, but only >0.4 μm [8], suggesting that smaller caliber targets may require additional mechanisms (e.g., increased adhesion) to ensure their myelination [24]. Therefore, given how readily oligodendrocytes can myelinate inert structures >0.4 μm *in vitro*, we predicted that large-caliber axons that are typically not myelinated may become ectopically myelinated when myelin supply exceeds axonal demand. We assessed myelination along large-caliber segments of Rohon-Beard axons, which are essentially never myelinated in control [3], despite residing adjacent to myelinated axons in the dorsal spinal cord and being accessible to oligodendrocytes. To visualize myelin along Rohon-Beard (RB) axons, we combined the Tg(isl1:Gal4; UAS:DsRed) animals, which have fluorescent RB neurons and axons [25], with Tg(mbp:EGFP-CAAX). We confirmed their very rare myelination in controls (1 myelin sheath associated with 76 RB neurons in 12 animals; see the example in Figure 3I) and found that this was not significantly increased in Skp2C25-treated animals (6 myelin sheaths with 96 RBs in 12 animals; Figure 3I; Mann-Whitney test; p = 0.205). These observations show that, although there may be occasional mistargeting of myelin to incorrect large-caliber axons, this does not happen readily. Together, our data indicate that myelin is targeted in a hierarchical fashion that prioritizes specific large-caliber axons and allows a surprising degree of myelination of cell bodies but rather stringently prevents myelination of incorrect axons, in line with previous studies that identified negative regulators of myelination [26]. It will be interesting in the future to determine where in the hierarchy of myelin targeting dendrites fall, given our previous findings of a somatodendritic inhibitor of myelination [4].

### Decreasing Oligodendrocyte Number Rescues Low Wild-Type Level of Cell Body Wrapping

In all analyses, we noted a low level of cell body wrapping in control animals, as we recently reported in mammals [4]. Based on our observations that the extent of myelin mistargeting correlated positively with the relative excess of oligodendrocytes, we predicted that animals with a reduced number of oligodendrocytes would exhibit little, if any, myelin mistargeting. To reduce oligodendrocyte number, we treated animals between 1.5 and 4 dpf with GANT61, an inhibitor of Gli1/2, the downstream effector of the sonic hedgehog signaling pathway essential for the generation of oligodendrocytes [27, 28, 29, 30]. This treatment reduced oligodendrocyte number below normal and did indeed reduce the already low number of myelinated cell bodies below that observed in wild-type (Figures 3A–3D). This result indicates the myelination is normally slightly biased toward slight overproduction, even at the risk of occasional, usually transient, mistargeting errors.

### Myelin Is Mistargeted to Neuronal Cell Bodies when Myelin Production by Oligodendrocytes Is Increased

**Figure 4:**
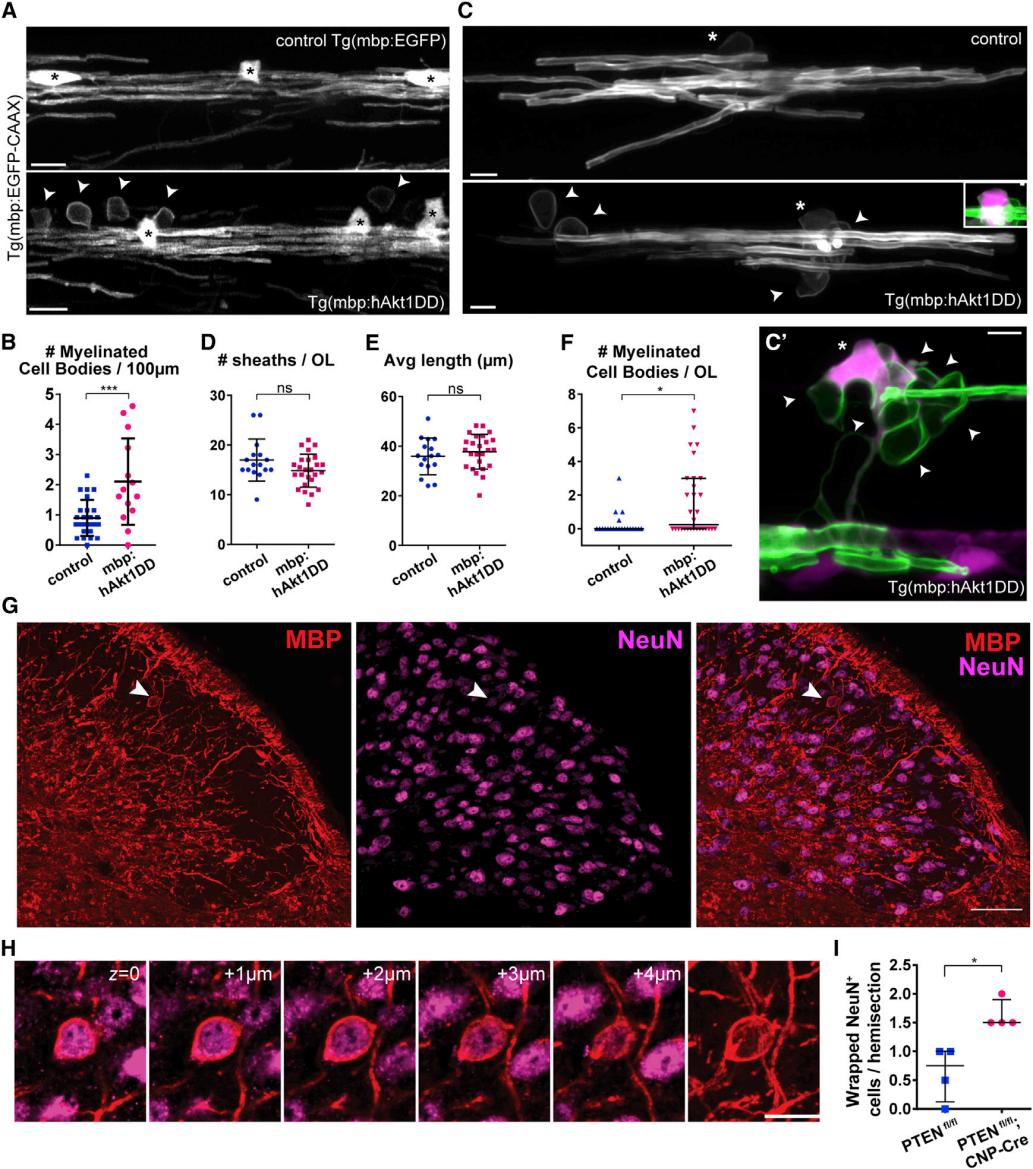
Increasing Myelin Production Induces Cell Body Wrapping in the Zebrafish and Mouse Spinal Cord (A) 5 dpf spinal cord of control and transgenic zebrafish overexpressing constitutively active human Akt1 in oligodendrocytes, Tg(mbp:hAkt1DD), in double oligodendrocyte and myelin reporter line (arrowheads, wrapped cell bodies; asterisks, oligodendrocytes). (B) The number of wrapped cell bodies is significantly increased in Tg(mbp:hAkt1DD) (p = 0.001; n = 25 control and n = 14 Tg(mbp:hAkt1DD); t test). (C–F) Individual oligodendrocytes (C; asterisks note their cell bodies; note cytoplasmic TagRFPt expression in Tg(mbp:hAkt1DD) cell in inset) have a similar myelin sheath number and length at 5 dpf, quantified in (D) (p = 0.077; n = 16 control and n = 25 Tg(mbp:hAkt1DD); t test) and (E) (p = 0.412; n = 16 control and n = 25 Tg(mbp:hAkt1DD); t test), but Tg(mbp:hAkt1DD) animals have more myelinated cell bodies (arrowheads), quantified in (F) (p = 0.011; n = 21 control and n = 30 Tg(mbp:hAkt1DD); Mann-Whitney test). (C’) Another example of an individual oligodendrocyte in Tg(mbp:hAkt1DD) showing ectopic myelination of multiple cell bodies (arrowheads; asterisk denotes oligodendrocyte cell body). (G) Immunostaining of myelin basic protein (red, MBP) and NeuN (magenta) in the dorsal horn of the cervical spinal cord. Arrowhead indicates a wrapped NeuN+ neuronal cell body. (H) Optical sections representing 1-μm increments in the axial plane. Rightmost panel is a maximum intensity projection of MBP. (I) Frequency of ectopic wrapping events is slightly increased in the cervical spinal cord of P30 PTEN^fl/fl^; CNP-Cre transgenic mice (p = 0.029; Mann-Whitney test; n = 4 animals per genotype; each animal quantified as the median of 6 pooled histological sections). All graphs display mean and SD, except for (F) and (I), which display median and interquartile range. The scale bars represent 10 μm (A), 5 μm (C and C’), 1 μm (inset C), 50 μm (G), and 20 μm (H).

Our results indicate that dysregulation of oligodendrocyte number relative to target axons can lead to the mistargeting of myelin. To test whether regulating the production of myelin by oligodendrocytes also influences its targeting, we took advantage of the observation that overexpressing a constitutively active form of Akt1 in oligodendrocytes increases myelination [31, 32, 33]. We first co-expressed constitutively active human Akt1 (hAkt1DD) in myelinating oligodendrocytes in zebrafish by generating the stable transgenic line Tg(mbp:hAkt1DD). In Tg(mbp:hAkt1DD) animals at 5 dpf we found a significant increase in the number of wrapped cell bodies (Figures 4A and 4B), with no changes to oligodendrocyte number (oligodendrocyte number per 100 μm of dorsal spinal cord: control 6 ± 2; n = 25; versus Tg(mbp:hAkt1DD) 7 ± 1; n = 14; p = 0.1; t test). Interestingly, we found that the number of myelin sheaths made per individual oligodendrocyte was similar in control and Tg(mbp:hAkt1DD) animals, as was myelin sheath length at these stages, but that each oligodendrocyte formed a significantly higher number of myelinated cell bodies (Figures 4C–4F). This analysis indicates that increasing myelin production can result in direct mistargeting of myelin to cell bodies.

We next asked whether increasing myelin production in mammals might also result in mistargeting. To test this, we increased Akt1 signaling in myelinating oligodendrocytes in mice by conditional ablation of PTEN, an inhibitor of the Akt1 pathway, using CNP-Cre. To assess whether this led to mistargeting of myelin to cell bodies, we performed MBP and NeuN immunostaining of cervical spinal cord sections to label myelin and neuron cell bodies, respectively (Figure 4G). At postnatal day 30 (P30), we found that the number of NeuN-positive neuronal cell bodies wrapped by MBP-positive immunostaining in the dorsal horn of the spinal cord of PTEN^fl/fl^; CNP-Cre animals was significantly increased compared to PTEN^fl/fl^ controls that do not express Cre (Figures 4G–4I). These data indicate that an imbalance of myelin supply and axonal demand can also lead to myelin mistargeting in mammals.

In sum, we have found that myelination by oligodendrocytes is normally biased toward slight overproduction *in vivo*, potentially to ensure robust myelination of the axons that need it, even if this results in a low level of (typically transient) myelin mistargeting. When interactions between axons and myelinating oligodendrocytes are dysregulated, and myelin is produced in excess of target axon demand, significant mistargeting to neuronal cell bodies can occur. Future studies will determine whether myelin targeting varies between species, distinct ages, or areas of the CNS; elucidate when during myelination extrinsic signals regulate myelin targeting: during the initial dynamic period of sheath formation and/or the subsequent period of refinement; and characterize the functional consequences of such mistargeted myelin on neuronal physiology. It will also be very important to determine whether compounds that promote oligodendrogenesis that are being considered as therapeutic candidates for the treatment of diseases of myelin [34] could result in myelin mistargeting and possible functional disruption. Our observations highlight how finely balanced myelination by oligodendrocytes is in the healthy nervous system and where vulnerabilities in the process of myelination may lie.

## STAR★Methods

### Key Resources Table

**Table:** 

REAGENT or RESOURCE	SOURCE	IDENTIFIER
**Antibodies**		

Chicken polyclonal anti-MAP2 (microtubule-associated protein 2), 1:500	Millipore	Cat# AB5543, RRID: AB_571049
Rat monoclonal anti-MBP (myelin basic protein), 1:200	Millipore	Cat# MAB386, RRID: AB_94975
Rabbit monoclonal anti-NeuN (neurogenin N), 1:1,000	Abcam	Cat# ab77315, RRID: AB_1566475
goat anti-rat AlexaFluor 488, 1:1,000	Life Technologies	Cat# A-11006, RRID: AB_2534074
goat anti-rabbit AlexaFluor 594, 1:1,000	Life Technologies	Cat# A-11037, RRID: AB_2534095
goat anti-chicken AlexaFluor 647, 1:1,000	Life Technologies	Cat# A-21449, RRID:AB_2535866

**Chemicals, Peptides, and Recombinant Proteins**		

Skp2-C25, Skp2 Inhibitor	Xcess Biosciences	Cat#: M60136-2 s
C646, p300/CBP inhibitor	Xcess Biosciences	Cat#: M60129-2 s, CAS: 328968-36-1
GANT61, GLI inhibitor	Enzo Life Sciences	Cat#: ALX-270-482-M001, CAS: 500579-04-4

**Experimental Models: Organisms/Strains**		

Zebrafish: kif1bp^st23^	[17]	ZFIN: ZDB-ALT-061207-8
Zebrafish: Tg(sox10:mRFP), vu234Tg	[35]	ZFIN: ZDB-ALT-080321-3
Zebrafish: Tg(olig2:EGFP), vu12Tg	[36]	ZFIN: ZDB-ALT-041129-8
Zebrafish: Tg(mbp:EGFP), ue1Tg	[16]	ZFIN: ZDB-ALT-120103-1
Zebrafish: Tg(mbp:EGFP-CAAX), ue2Tg	[16]	ZFIN: ZDB-ALT-120103-2
Zebrafish: Tg(cntn1b:mCherry), ue3Tg	[15]	ZFIN: ZDB-ALT-140610-5
Zebrafish: Tg(elavl3:Gal4VP16), ue8Tg	[37]	ZFIN: ZDB-ALT-170419-1
Zebrafish: Tg(NBT:DsRed) in text, synon. Tg(Xla.Tubb:DsRed), zf148Tg	[38]	ZFIN: ZDB-ALT-081027-2
Zebrafish: Tg(isl1:Gal4-VP16,UAS:dsRed), zf234Tg	[25]	ZFIN: ZDB-ALT-110520-2
Zebrafish: Tg(mbp:tagRFPt-2A-hAkt1DD), abbreviated as Tg(mbp:hAkt1DD) in text	this paper	N/A
Mouse: Pten^fl/fl^	Dr. Eliott Sherr	RRID:IMSR_JAX:006440.
Mouse: CNP-Cre	Dr. Klaus Armin-Nave	RRID:MGI:3051754

**Oligonucleotides**		

Morpholino: MO5-notch1a, GTAGTGTTAAACTGTTACCTTGTGC	Gene Tools	ZFIN: ZDB-MRPHLNO-070622-1
Primers for kif1bp^st23^, PTEN^fl/fl^, CNP-Cre genotyping and for recombinant DNA generation, see Table S1	This paper, see Table S1.	N/A

**Recombinant DNA**		

pTol2- EGFP-UAS-tagRFPt-cntn1a	this paper	N/A
pTol2- mbp:tagRFPt-2A-hAkt1DD	this paper	N/A
pTol2- mbp:EGFP-CAAX, *syn. Tg(mbp:EGFP-CAAX)*	[16]	ZFIN: ZDB-TGCONSTRCT-120103-3
tol2kit	[39]	http://tol2kit.genetics.utah.edu/index.php/Main_Page

**Software and Algorithms**		

GraphPad Prism	GraphPad Software	RRID:SCR_015807
Fiji	[40]	RRID:SCR_002285
Adobe Illustrator	Adobe	RRID:SCR_010279

### Contact for Reagent and Resource Sharing

Further information and requests for resources and reagents should be directed to and will be fulfilled by the Lead Contact, David Lyons (david.lyons@ed.ac.uk).

### Experimental Model and Subject Details

#### Zebrafish Lines and Maintenance

All zebrafish were maintained under standard conditions [41, 42] in the Queen’s Medical Research Institute CBS Aquatics facility at the University of Edinburgh. Studies were carried out with approval from the UK Home Office and according to its regulations, under project licenses 60/8436 and 70/8436. Adult animals were kept in a 14 hours light and 10 hours dark cycle. Embryos were kept at 28.5°C in 10mM HEPES-buffered E3 Embryo medium or conditioned aquarium water with methylene blue. Embryos were staged according to [43], and analyzed between 2-8 dpf, well before the onset of sexual differentiation in zebrafish.

The following existing mutant and transgenic lines were used: *kif1bp*^st23^ [17]; Tg(sox10:mRFP) [35]; Tg(olig2:EGFP) [36]; Tg(mbp:EGFP) [16]; Tg(mbp:EGFP-CAAX) [16]; Tg(cntn1b(5kb):mCherry) [15]; Tg(elavl3:Gal4VP16) [37]; Tg(Xla.Tubb:DsRed) referred to as Tg(NBT:DsRed) in text [38]; Tg(isl1:Gal4-VP16,UAS:RFP) [25]. The line Tg(mbp:tagRFPt-2A-hAkt1DD) was generated in this study (see details below). *kif1bp*^st23^ animals were genotyped as before [18], using primers st23F2 and st23R2 (Table S1) followed by digestion of the PCR product with restriction enzyme NmuCI, which cleaves the WT PCR product into 228bp and 22bp fragments, but not the mutant product. In some experiments, *kif1bp*^st23^ homozygous mutants were identified by direct visualization of an incompletely grown posterior lateral line nerve in Tg(mbp:EGFP-CAAX) or Tg(sox10:mRFP) larvae. Mutants were compared to wild-type-siblings (“WT”). Throughout the text including figures, ‘Tg’ denotes a stable, germline inserted transgenic line.

#### Mouse lines

All mice used in this study were housed and handled with the approval of the University of California, San Francisco Institutional Animal Care and Use Committee (IACUC). Male and female mice group housed in a 12-hour light/dark cycle with *ad libitum* access to food and water and aged to postnatal day 30 were used in all experiments. Pten^fl/fl^;CNP-Cre mice were assigned to the experimental group, while Pten^fl/^fl;CNP-Cre littermate controls were assigned to the control group. Pten^fl/fl^ mice were a generous gift from Dr. Eliott Sherr, and CNP-Cre mice were a generous gift from Dr. Klaus Armin-Nave [44, 45]. Pten^fl/fl^ and CNP-Cre were genotyped via standard PCR, using primers oIMR9554/oIMR9555 and Puro3/E3sense/EcoIN2, respectively (Table S1).

### Method Details

#### Generation of zebrafish with supernumerary Mauthner axons

We generated animals with supernumerary Mauthner neurons as previously described [16], by injection of 500pg of a morpholino targeting a *notch1a*-specific splice junction [46] in fertilized eggs between 1-8 cell stage. Successful supernumerary Mauthner neuron induction was identified by visualizing supernumerary Mauthner axon myelin sheaths with the Tg(mbp:EGFP-CAAX) transgene.

#### Generation of EGFP-UAS-tagRFPt-cntn1a axonal myelination reporter

We previously described the axonal reporter EGFP-cntn1a, which labels unmyelinated segments [3]. We replaced the EGFP coding sequence with that of tagRFPt, by ligating the tagRFPt cDNA amplified with primers tagRFPt-F and tagRFPt-nostop-R (Table S1) to a Gateway 3′-entry vector containing the original EGFP-cntn1a cDNA, amplified with primers cntn1a-F and cntn1a-signal-R (Table S1), which amplify the whole plasmid except for the EGFP cDNA. We confirmed correct cloning of tagRFPt after the cntn1a signal peptide by Sanger sequencing. We then recombined this 3E-tagRFPt-cntn1a with a 5′-entry vector containing the EGFP coding sequence in the reverse orientation to that of tagRFPt-cntn1a, a middle-entry vector containing 5 Gal4 binding sites (upstream activating sequences, UAS) flanked on both sides by E1b minimal promoters in opposite orientations, and destination vector pDestTol2pA2 from the tol2kit [39], using LR Clonase II Plus. This generated the bidirectional, Gal4-dependent construct pTol2-EGFP-UAS-tagRFPt-cntn1a, where the EGFP and tagRFPt-cntn1a sequences are arranged in a ‘Janus’ configuration, facing ‘away’ from each other, leading to efficient expression of both reporters in individual cells [47, 48].

#### Generation of Tg(mbp:tagRFPt-2A-hAkt1DD)

To generate a constitutively-active form of human Akt1 (hAkt1), we first mutagenized the coding sequence of human Akt1 in a Gateway middle-entry vector, ME-myrhAkt1 (kind gift of Dirk Sieger), to generate the 308D and 473D point mutations (Akt1DD), previously described to mimic the phosphorylated activated state of Akt1 [49], with primers Akt1-308D-F, Akt1-308D-R, Akt1-473D-F and Akt1-473D-R (Table S1). We verified successful mutagenesis by Sanger sequencing. We then used recombinant PCR [50, 51] to generate the coding sequence for tagRFPt-2A-hAkt1DD, wherein the short self-cleaving 2A peptide yields a one-to-one stoichiometry of tagRFPt and hAkt1DD, enabling us to identify the cells expressing hAkt1DD by tagRFPt expression. To do this, we amplified tagRFPt (without a stop codon) with primers attB1-tagRFPt-F and tagRFPt-2A-R; and amplified hAkt1DD (with a stop codon) with primers 2A-Akt1-F and attB2R-Akt1-R. We then generated the full length attB1-tagRFPt-2A-hAkt1DD-attB2R using primary PCR products as templates and the outer flanking primers containing attB sites. We recombined the full length PCR product with pDONR221 to generate middle entry vector ME-tagRFPt-2A-Akt1DD using BP Clonase, whose sequence we verified by Sanger sequencing. We then recombined our previously described 5′-entry vector 5E-mbp [16], with ME-tagRFPt-2A-Akt1DD and 3E-polyA and pDestTol2pA2 from the tol2kit, using LR Clonase II Plus. This generated the pTol2-mbp:tagRFPt-2A-hAkt1DD construct, which yielded tagRFPt-expressing oligodendrocytes when transiently expressed in injected zebrafish embryos. We established a germline-inserted stable transgenic line by screening for founders derived from co-injection of 1nL of 10 ng/μL pTol2-mbp:tagRFPt-2A-hAkt1DD plasmid DNA with 50 ng/μl *tol2* transposase mRNA. In this line, all oligodendrocytes express tagRFPt and thus hAkt1DD. For brevity, we refer to this line as Tg(mbp:hAkt1DD) in the main text.

#### Single cell labeling

To mosaically label oligodendrocytes, we injected fertilized eggs with 1nL of 10ng/μL pTol2-mbp:EGFP-CAAX plasmid DNA and 50ng/μl *tol2* transposase mRNA. Animals were screened at 4dpf for isolated oligodendrocytes. To express the myelination reporter in individual reticulospinal axons, Tg(elavl3:Gal4VP16) fertilized eggs were injected with 1nL of 10ng/μL pTol2-EGFP-UAS-tagRFPt-cntn1a plasmid DNA and 50ng/μl *tol2* transposase mRNA. Animals were screened at 4dpf for EGFP+ and tagRFP-cntn1a+ reticulospinal axons, descending from labeled neurons in the hindbrain, located in the ventral tract of the spinal cord and identified by their typical collateral branches. Typically only one cell per animal was imaged; when more than one cell per animal was imaged, data were averaged per individual animal.

#### Transmission electron microscopy

Tissue was prepared for TEM as previously described [18, 52]. Zebrafish embryos were terminally anaesthetised in tricaine and incubated, with microwave stimulation, first in primary fixative (4% paraformaldehyde 2% glutaraldehyde in 0.1M sodium cacodylate buffer) and then in secondary fixative (2% osmium tetroxide in 0.1M sodium cacodylate/imidazole buffer). Samples were then stained en bloc with a saturated uranyl acetate solution and dehydrated in an ethanol series and acetone, both with microwave stimulation. Samples were embedded in EMbed-812 resin (Electron Microscopy Sciences), and sectioned using a Reichert Jung Ultracut Microtome. Sections were cut at comparable somite levels by inspection of blocks under a dissection microscope, and stained in uranyl acetate and Sato lead stain. TEM images were taken with a Phillips CM120 Biotwin TEM.

#### Drug Treatments

We sought to treat zebrafish embryos after neurogenesis and axonal outgrowth, but during a period that encompasses all stages of oligodendrocyte development (from specification of precursors to myelination), in order to bias our treatments to affect oligodendrocyte-lineage cells. To this end, we treated zebrafish embryos between 1.5-2dpf and 4dpf. By 2dpf, for example, the majority of motor neurons have been born in the developing zebrafish spinal cord [53]; and most reticulospinal axons have reached the posterior end of the spinal cord [18]; whereas oligodendrocyte precursor specification is just starting (between 36hpf and 48hpf) [18, 35]. We treated embryos by bath-application of Skp2C25 (2 μM), C646 (2 μM), GANT61 (10 μM from 1.5dpf), all in 1% DMSO, dissolved in HEPES-buffered embryo medium, or with only 1% DMSO in HEPES-buffered embryo medium as control. Zebrafish larvae tolerated these treatments well; we selected morphologically normal and healthy animals at 4dpf for subsequent analyses.

#### Mouse tissue collection and immunostaining

Spinal cord tissue was collected from P30 mice using standard techniques. Briefly, after transcardial perfusion with PBS and then 4% PFA (Electron Microscopy Tools), the spinal cord was dissected out and post-fixed in 4% PFA overnight at 4C and cryoprotected by incubation in 30% sucrose in PBS. Coronal sections were cut in 30-mm thick slices from the cervical spinal cord (C5–C8) using a freezing microtome (Microm HM 450 and KS 34, Thermo Scientific). Sections were immunostained using standard methods. In brief, free-floating sections were permeabilized in 20% normal goat serum (NGS) (Sigma-Aldrich) plus 0.1% Triton X-100 (Sigma-Aldrich) in, followed by an overnight incubation at 4C with primary antibodies in permeabilization solution. Sections were washed in PBS and incubated with secondary antibodies for 4 hours at room temperature. Subsequently, sections were washed in PBS and then water, mounted onto glass slides, air-dried, and coverslipped with fluorescent mounting medium (Dako).

Primary antibodies against microtubule-associated protein 2 (chicken anti-MAP2, 1:500, Millipore), MBP (rat anti-MBP, 1:200), and NeuN (rabbit anti-NeuN, 1:1,000, Abcam) were used for immunostaining. Secondary antibodies were Alexa Fluor raised in goat against rat, rabbit, or chicken in the following wavelengths: 488, 594, and 647 (1:1,000, Life Technologies), and nuclei were stained with DAPI.

#### Live imaging

We live-imaged embryos by anesthetising them with tricaine and mounting them on their side in coverslips, embedded in 1.5% low melting point agarose. Z stacks were acquired using confocal microscopes Zeiss LSM710 or LSM880 equipped with Airyscanner, or a Zeiss AxioImager Z1 equipped with an Apotome2 structured illumination unit; and a 20X objective (Zeiss Plan-Apochromat 20x dry, NA = 0.8), a water-immersion 63x objective (Zeiss C-Apochromat 63x, NA = 1.2) or an oil-immersion 63x objective (Zeiss Plan-Apochromat 63x, NA = 1.4). Z stacks were acquired with a z-step between 0.25-2 μm according to the experiment. For *kif1bp*^st23^ analyses, images of the anterior region of the spinal cord were acquired by aligning the edge of the visual field to the urogenital opening somite, and the posterior region by aligning the visual field to the last somite in the spinal cord. For manipulations other than *kif1bp*^st23^, which are not expected to have a region-specific effect, we focused on the middle spinal cord, by aligning the center of the visual field to the urogenital opening. The following lengths of spinal cord were sampled in the following data-sets: Figure 1A:448 μm; Figure 1G 304 μm or as in Figure 3A detailed below; Figure 2H: 236 μm; Figure 3I: 234 μm; Figure 4A: 434 μm. Timelapses of Tg(olig2:EGFP); Tg(sox10:mRFP) were acquired as before [18]. For drug treatments shown in Figure 3A, embryos were loaded and oriented for imaging using an LPSampler and VAST BioImager fitted with a 600μm capillary (Union Biometrica). We imaged the whole length of the embryo (> 3mm) with a Zeiss Axio Examiner D1 equipped with a CSU-X1 spinning confocal scanner, a Zeiss AxioCam 506 m CCD camera and a Zeiss C-Plan-Apochromat 10x 0.5NA objective, by tiling 5 z stacks per embryo with a z-step of 2-3 μm. Data were presented as density per 100 μm of spinal cord length when necessary to pool datasets with different lengths sampled, or normalized by dividing each data point by its session’s control average, and graphed as % of the control average (e.g., Figures 1H and 1I).

#### Image processing and analysis

Image processing and analysis was performed in Fiji (a distribution of ImageJ). Figure panels were produced using Fiji and Adobe Illustrator CS8. For figures, maximum-intensity projections of Z stacks were made, and a representative x-y area was cropped. All zebrafish images and movies represent a lateral view of the spinal cord, anterior to the left and dorsal on top. For most images, processing included only global change of brightness and contrast; further processing and analysis is as follows.

##### Cell counts

a grid was superimposed and all EGFP-filled cell bodies (oligodendrocytes) and EGFP-CAAX-surrounded cell bodies not filled with cytoplasmic EGFP (myelinated cell bodies) were counted through z stacks encompassing the depth of the spinal cord, using the cell counter plugin in Fiji. An approach based on unbiased stereological methods was used to ensure that each cell was only counted once. For drug-treated animals (Figure 3A), we counted all dorsal oligodendrocytes and dorsal myelinated cell bodies along the entire length of the spinal cord in maximum-intensity projections, while blinded to treatment conditions. When data from multiple experimental sessions were pooled, to graph normalized data, we divided each data point by its session’s control (WT or DMSO-treated) average, and graphed each data point as a percentage of the control average (Figures 3B–3D).

##### Individual oligodendrocyte morphology

for *kif1bp* analysis, oligodendrocytes isolated in the field of view with cell bodies clearly positioned in the ventral or dorsal tracts were considered, and the number of sheaths, their length and their location on dorsal, medial, ventral or motor tracts analyzed throughout the z stack. For Tg(mbp:hAkt1DD) analysis, only dorsal oligodendrocytes were considered. Analyses were performed while blinded to genotype.

##### Electron micrographs

the Photomerge tool in Adobe Photoshop was used first for automatic registration and tiling. Axons containing identifiable neurofilament cross-sectional profiles (10nm), with a roughly circular profile and located in the white matter were traced and measured in Adobe Photoshop or Fiji and their area was used to determine the corresponding diameter. Tracing was performed while blinded to genotype.

##### Individual reticulospinal axons

EGFP+ axons located in the ventral spinal cord with at least two collateral branches and one myelin sheath (tagRFPt-contactin1a gap) were considered, and axonal length and the number and length of tagRFPt-contactin1a gaps analyzed throughout the Z stack. Analysis was performed while blinded to genotype.

##### Very dorsal myelin sheaths

all myelin sheaths that were located above the dorsal most myelinated axon in the dorsal tract of the spinal cord of Tg(mbp:EGFP-CAAX) transgenic animals were counted and measured through the z stack, while blinded to drug treatment.

##### Time-lapse

Z stacks were maximum intensity-projected for each time point; the olig2:EGFP channel was registered between time points using the ‘Register virtual stack slices’ plugin in Fiji, and the ‘Transform virtual stack slices’ plugin was used to apply the same transformations to the sox10:mRFP channel.

#### Mouse spinal cord image acquisition and analysis

Images were acquired using an Andor Borealis CSU-W1 spinning disk confocal system. Stitched reconstructions of spinal cord hemisections were generated in Fiji, and the incidence of wrapped cell bodies (as determined by tight apposition of NeuN+ cell bodies with MBP throughout individual planes in the z stack) was quantified by a blinded observer. The region of the dorsal horn containing NeuN^+^ cell bodies (approximately 8,000 – 9,000 μm^2^) from six cervical spinal cord (C5 – C8) hemisections were analyzed per mouse, and the pooled medians of hemisections from either Pten^fl/fl^/CNP-Cre or Pten^fl/fl^ animals were compared via a Mann-Whitney U test.

### Quantification and Statistical Analyses

All graphs and statistical tests were carried out using GraphPad Prism. All data were averaged per biological replicate (N represents number of animals). Data was tested for normal distribution using D’Agostino-Pearson omnibus test and compared between groups using a two-tailed unpaired Student’s t test; and the distribution of all data was also compared using the Mann-Whitney U test. We considering a difference significant when p < 0.05, and all comparisons maintained the same significance using the t test or Mann-Whitney test. Throughout the figures, we indicate p values as follows: no indication or ‘ns’ p > 0.05, ‘^∗^’ p < 0.05, ‘^∗∗^’ p < 0.01, ‘^∗∗∗^’ p < 0.001. Indicated p values are from t test comparison in all graphs where datasets passed the normality test; when data was clearly not normally distributed (Figures 2D, 4F, and 4K), we indicate the result of the Mann-Whitney U test. Throughout the figures, error bars illustrated mean ± standard deviation (SD), except where noted otherwise. For comparisons of myelinated axon distribution, p values were corrected for multiple comparisons using the Holm-Sidak method. Statistical tests were performed on original data, and p value results were not changed by transformation of data, i.e., when divided by control average or expressed as density (number per 100μm length of spinal cord). Details of statistical test used, precise p value and *N* values for each comparison are detailed in Figure Legends.

## Supplemental Information

Document S1. Figure S1 and Table S1

Movie S1. 3D View of Wrapped Neuron Somas in kif1bp Mutants, Related to Figure 1 Green is Tg(mbp:GFP-CAAX) and Magenta is Tg(cntn1b:mCherry).

Movie S2. Time-Lapse Imaging of Tg(olig2:EGFP); Tg(sox10:mRFP) Oligodendrocyte-Lineage Cells Wrapping Cell Bodies as They Differentiate in a *kif1bp* Mutant, Related to Figure 1 Blue arrowhead points to a myelin sheath being formed concomitantly with wrapping of cell bodies (magenta arrowheads).

Document S2. Article plus Supplemental Information
