# Supplementary material for: Myelination of Neuronal Cell Bodies when Myelin Supply Exceeds Axonal Demand
Source: Curr Biol. 2018 Apr 23;28(8):1296–1305.e5. doi: 10.1016/j.cub.2018.02.068 (PMC5912901; doi:10.1016/j.cub.2018.02.068)
Supplement: Document S1. Figure S1 and Table S1 [file mmc1.pdf]

Current Biology, Volume *28*

Supplemental Information

Myelination of Neuronal Cell Bodies when Myelin Supply Exceeds Axonal Demand

Rafael G. Almeida, Simon Pan, Katy L.H. Cole, Jill M. Williamson, Jason J. Early, Tim Czopka, Anna Klingseisen, Jonah R. Chan, and David A. Lyons

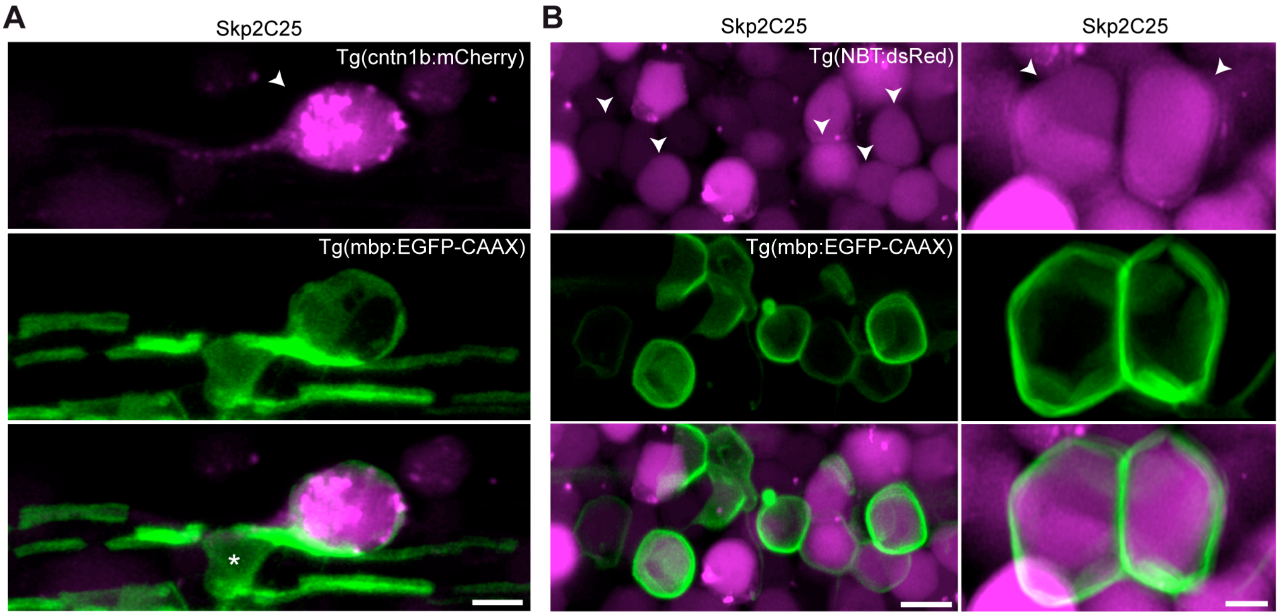

Figure S1. **Wrapping of cell bodies in Skp2C25 treated animals, related to Figure 3. (A).** Skp2C25-treated double transgenic animals with a fluorescently labelled Rohon Beard neuron Tg(cntn1b:mCherry) wrapped in myelin by a Tg(mbp:EGFP-CAAX) expressing myelinating oligodendrocyte. **(B)** Skp2C25-treated double transgenic animals with fluorescently labelled neurons Tg(NBT:dsRed) wrapped in myelin. Right shows a second example at higher magnification.

Table S1 – Primers used in this study, related to STAR Methods

Primer	Sequence (5'-3')
**st23F2**	AAA ACG ACC AAC TGT GCC TA
**st23R2**	ACA GTC AAA CAC CAG ATC GAA AGT CA
**tagRFPt-F**	ATG GTG TCT AAG GGC GAA GAG CTG
**tagRFPt-nostop_R**	CTT GTA CAG CTC GTC CAT GCC ATT AAG
**cntn1a-F**	CCG GTG TTC GAG GAG CAG CCG C
**cntn1a-signal-R**	GGT CCC GAA GCC TGC AGA CTC ACC
**Akt1-308D-F**	GTG CCA CCA TGA AGG ACT TTT GCG GCA C
**Akt1-308D-R**	CGT CCT TGA TCC CCT CCT TGC ACA GCC
**Akt1-473D-F**	CAC TTC CCC CAG TTC GAC TAC TCG GCC AGC G
**Akt1-473D-R**	GGG CCT GCG CTC GCT GTC CAC ACA CTC CAT
**attB1-tagRFPt-F**	GGG GAC AAG TTT GTA CAA AAA AGC AGG CTG CCG CCA CCA TGG TGT CTA AGG GCG AAG AGC
**tagRFPt-2A-R**	GTC TCC TGC TTG CTT TAA CAG AGA GAA GTT CGT GGC TCC GGA TCC CTT GTA CAG CTC GTC CAT GCC A
**2A-Akt1-F**	GCC ACG AAC TTC TCT CTG TTA AAG CAA GCA GGA GAC GTG GAA GAA AAC CCC GGT CCT ATG AGC GAC GTG GCT ATT GTG AAG GAG
**attB2R-Akt1-R**	GGG GAC CAC TTT GTA CAA GAA AGC TGG GTT CAG GCC GTG CCG CTG GC
**oIMR9554**	CAA GCA CTC TGC GAA CTG AG
**oIMR9554**	AAG TTT TTG AAG GCA AGA TGC
**Puro3**	CAT AGC CTG AAG AAC GAG A
**E3sense**	GCC TTC AAA CTG TCC ATC TC
**EcoIN2**	GAT GGG GCT TAC TCT TGC
